# Unanticipated Difficult Intubation in a Child Due to a Vallecular Cyst: A Case Report

**DOI:** 10.7759/cureus.111724

**Published:** 2026-06-29

**Authors:** Nand Kishore Joshi, Revanth B Challa, Reshma K B, Mangesh Mulaokar, Barkha D Agrawal, Ashutosh Kumar

**Affiliations:** 1 Anaesthesiology, All India Institute of Medical Sciences, Nagpur, Nagpur, IND

**Keywords:** bougie-guided intubation, difficult intubation, pediatric airway, supraglottic airway failure, vallecular cyst, videolaryngoscopy

## Abstract

Vallecular cysts are rare mucous-retention lesions that can cause potentially fatal airway obstruction during anesthesia while remaining clinically silent. We describe the case of a five-year-old girl who was scheduled for orthopedic surgery and experienced an unexpectedly challenging intubation as a result of an undetected vallecular cyst. A cystic mass blocking the laryngeal inlet was discovered by direct laryngoscopy, but mask ventilation was still effective. Visualization was enhanced by videolaryngoscopy, and a bougie-guided endotracheal intubation was successful. Airway anatomy was restored by aspirating a vallecular cyst that was discovered during intraoperative ENT evaluation, and the patient was extubated successfully. Later, definitive marsupialization was performed before planned orthopedic surgery, and this time, the intubation was smooth. This case highlights the possibility that vallecular cysts could make supraglottic devices unsuitable, necessitating the use of videolaryngoscopy, fibreoptic techniques, or early surgical backup.

## Introduction

One major issue that significantly increases perioperative morbidity and mortality during anesthesia is difficult airway management, particularly when it is unexpected. Undesirable consequences, such as airway trauma, cerebral hypoxia, and cardiopulmonary arrest, may result from a delay in diagnosing and treating such cases [1,2]. The unique anatomical features, congenital abnormalities, co-occurring diseases, and scarcity of appropriately sized airway devices in the pediatric population make intubation challenging [2,3]. Children are less tolerant of ventilation disruptions than adults because they consume more oxygen and have a smaller oxygen reserve. They are therefore vulnerable to rapid desaturation, which can lead to bradycardia very quickly [4]. Vallecular cysts, rare lesions with an incidence of ~1.8 per 100,000, are more common in infants and young children [5]. They are a recognized cause of unexpected airway emergencies during anesthesia, highlighting the need for vigilance even when preoperative airway assessment is unremarkable. We describe a case of a vallecular cyst that was identified during the induction of general anesthesia, which obscured the laryngeal inlet, causing difficulty in tracheal intubation.

## Case presentation

A five-year-old female child (weighing 15 kg) was posted for an Ilizarov procedure for congenital right-sided fibula hemimelia. The child was diagnosed with right lower limb deformity antenatally and had an uneventful birth history. All milestones were achieved as per age, and she used an artificial limb for mobilization. The parents denied any previous surgical history. Preoperative airway assessment revealed adequate mouth opening, full neck mobility, and no external anatomical abnormalities. Formal Mallampati grading was not feasible due to the patient's young age and limited cooperation. As there were no clinical features suggestive of an upper airway pathology, no preoperative airway imaging was performed.

On the day of surgery, the patient was taken into the operating room, and standard monitors (including pulse oximeter, noninvasive blood pressure, and electrocardiography) were attached. An intravenous line was already in situ. The patient was induced with fentanyl 2 µg/kg and propofol 2 mg/kg intravenously. After confirming the adequacy of mask ventilation, an intravenous injection of atracurium 0.5 mg/kg was administered. During laryngoscopy, with a Macintosh blade no. 2, a cystic mass was visualized at the base of the tongue, abutting the vision of the vocal cord.

**Figure 1:**
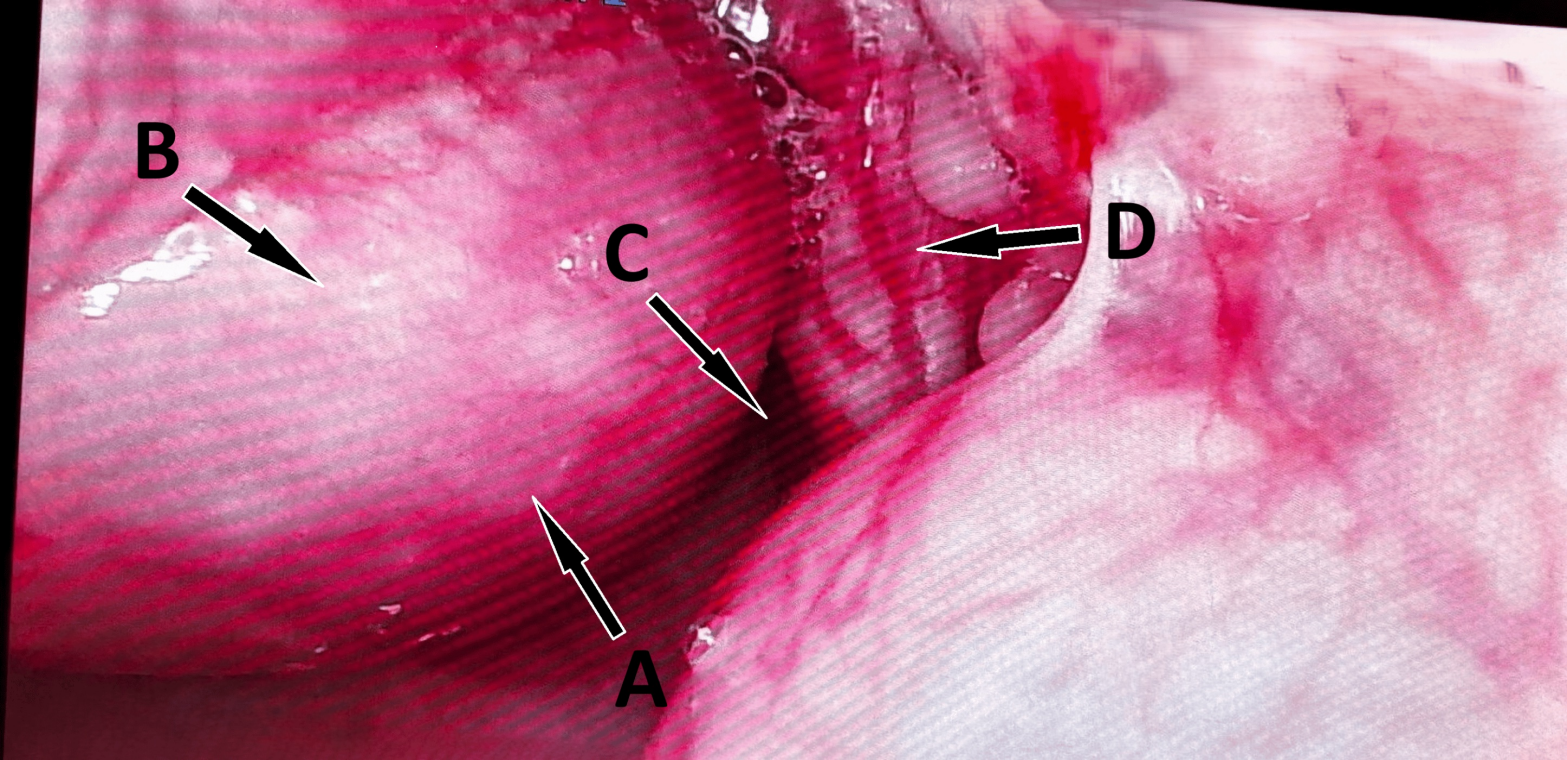
Videolaryngoscopic view. The image confirms the presence of a vallecular cyst (A) with a smooth, glistening surface (B). The cyst prolapses into and obliterates the glottic inlet (C), with the vocal cords (D) partially visible.

Mask ventilation was continued. SpO₂ dropped to 95% from 99% with a transient rise in heart rate to 130 beats/minute from 102 beats/minute, consistent with early hypoxemia and a sympathoadrenal stress response. A call for help was placed. A second attempt at laryngoscopy was performed using a videolaryngoscope. Videolaryngoscopy vision confirmed a round, regular cystic mass at the base of the tongue, falling into the vallecula, and a diagnosis of a vallecular cyst was evoked. The cord was visualized below the cyst using videolaryngoscopy (Figure 1). A cuffed endotracheal tube no. 5.0 was railroaded along a bougie and was fixed at an angle of mouth. After confirming bilateral air entry, mechanical ventilation was started. Following successful endotracheal intubation and initiation of mechanical ventilation, SpO₂ improved to 100% within approximately two minutes, and hemodynamic stability was fully restored. Injection hydrocortisone 2 mg/kg intravenously was administered prophylactically for potential airway edema secondary to prolonged instrumentation.

**Figure 2:**
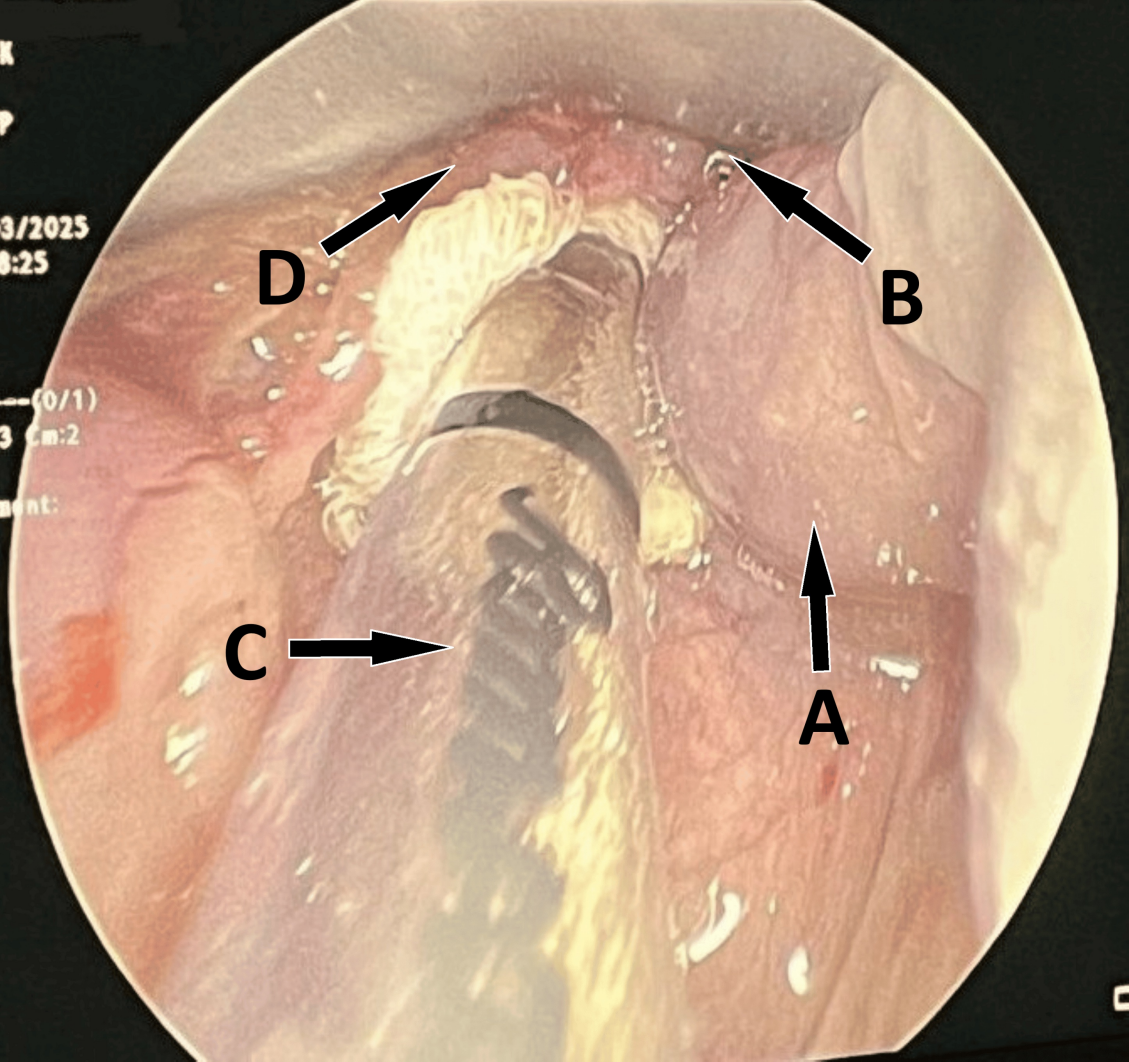
Endoscopic view. The image confirms the presence of a vallecular cyst (A) arising from the vallecula (B). The cuffed endotracheal tube (no. 5.0) shaft (C) is seen passing alongside the cyst. The epiglottis (D) is partially visible, displaced laterally by the cyst bulk.

**Figure 3:**
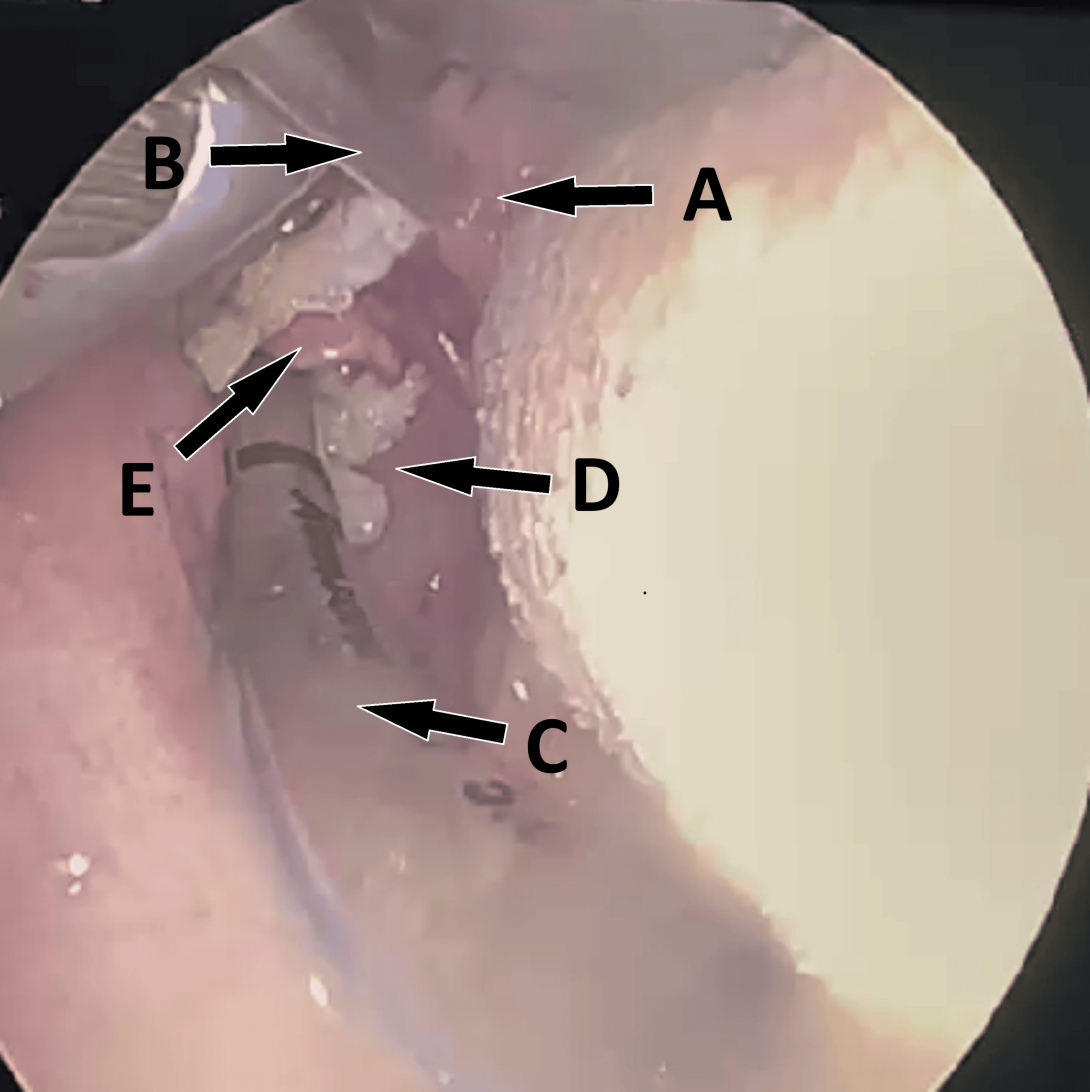
Endoscopic view after aspiration of the vallecular cyst. The collapsed cyst wall (A) is seen following aspiration of clear serous contents. The ENT aspiration instrument (B) is in position at the cyst base. The endotracheal tube (C) remains secured in situ throughout the procedure. The cleared glottic corridor (D) is becoming progressively visible, and the epiglottis (E) is now clearly identifiable as the cyst bulk is decompressed.

On table, ENT consultation was sought, and endoscopic evaluation confirmed the presence of a vallecular cyst (Figure 2, Video 1), following which endoscope-guided aspiration of the vallecular cyst was performed (Video 2). The aspirate was clear, serous, and non-hemorrhagic. Once the cyst was aspirated, the glottic area was visualized along with the endotracheal tube (Figure 3, Video 3). The proposed orthopedic surgery was abandoned and suggested for planned marsupialization of the vallecular cyst in the next sitting, as there was a chance of recollection of contents. The parents were counselled regarding the same. Once spontaneous attempts of breathing started, reversal of residual neuromuscular blockade was done, the patient was allowed to recover in the left lateral position, and was extubated once laryngeal reflexes were present.

**Video 1:** Endoscopic view confirming the presence of a vallecular cyst.

**Video 2:** Endoscope-guided aspiration of the vallecular cyst.

**Video 3:** Endoscopic glottic view after aspiration of the cyst with the endotracheal tube in situ.

The patient was transferred to the postoperative care unit (PACU) with oxygen supplementation with a Hudson mask. In the PACU, the patient was conscious, oriented, following simple commands, and telling her name. During retrospective interrogation, the parents denied any history, such as dysphagia, dyspnea, and stridor, suggestive of a vallecular cyst.

**Figure 4:**
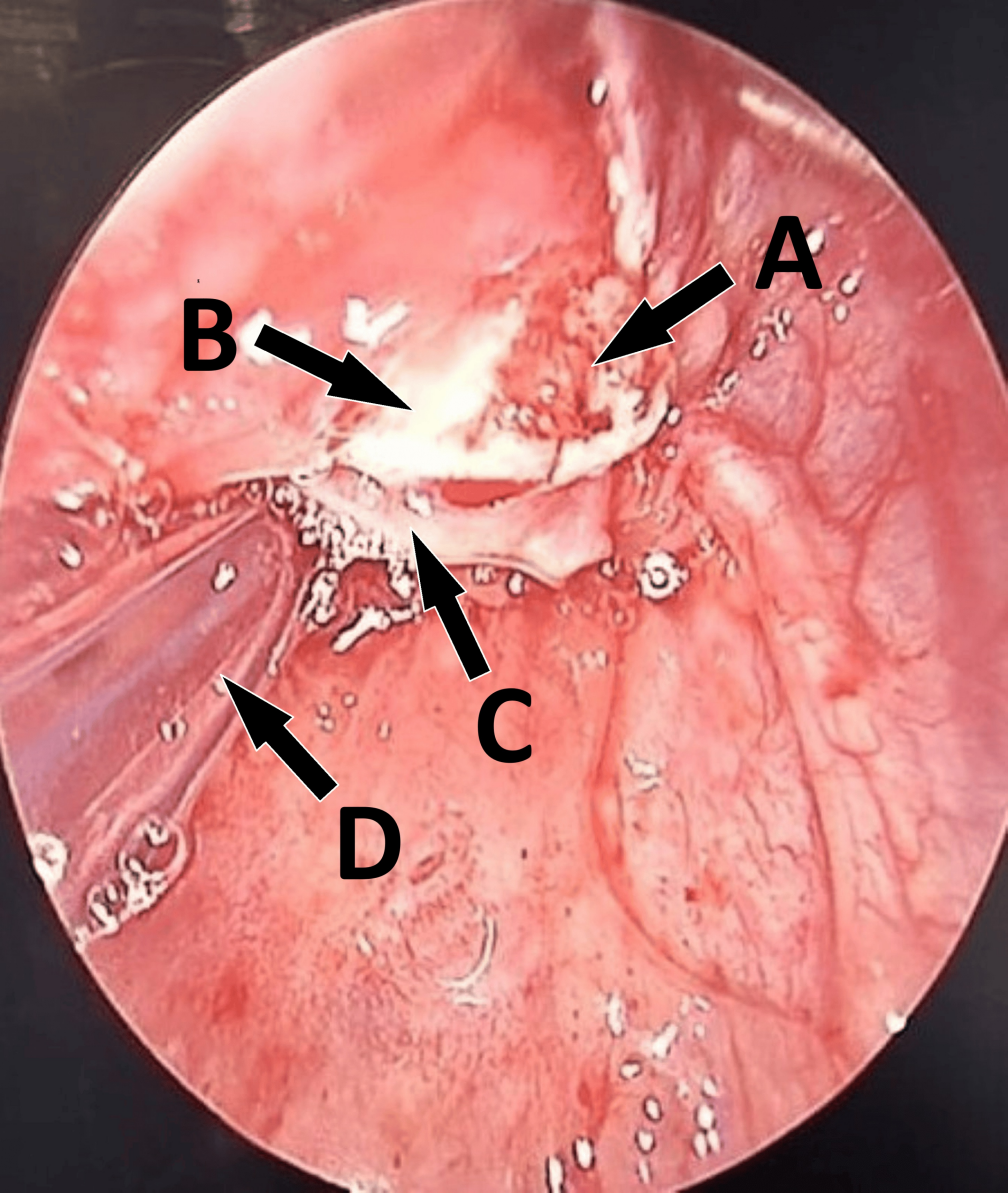
Post-marsupialization endoscopic view demonstrating restoration of normal laryngeal anatomy. The marsupialization bed (A) and excised cyst wall (B) are visible superiorly at the site of the former vallecular cyst. The epiglottis (C) is now visible confirming restoration of normal supraglottic anatomy, with the endotracheal tube in situ (D) secured throughout the marsupialization procedure.

After three weeks, the child was posted for the proposed orthopedic surgery along with marsupialization of the vallecular cyst. At this time, intubation was uneventful using a videolaryngoscope. She underwent definitive marsupialization (Figure 4) followed by orthopedic surgery. After completion of the surgery, the child was extubated and shifted to the room.

## Discussion

Vallecular cysts are rare but clinically significant airway lesions that may lead to unexpected difficulty in securing the airway, particularly in pediatric patients. These cysts result from mucous retention due to obstruction of submucosal gland ducts and have been described under various names, including mucous-retention cyst, ductal cyst, congenital cyst, and epiglottic cyst. Larger vallecular cystic lesions can result in feeding difficulties, stridor, dysphagia, or voice changes, though many patients remain asymptomatic. Crucially, they may remain clinically silent until anesthesia is induced, which could result in an acute airway crisis, as seen in our case. When pharyngeal muscle tone is lost, vallecular cysts can obstruct the laryngeal inlet by prolapsing backward, which is the main anesthetic concern.

Children are particularly at risk because of their increased oxygen consumption, decreased functional residual capacity, and quick desaturation during apnea. Maintaining oxygenation and preventing repeated traumatic attempts must therefore be given top priority when an unsuspected cyst is found [3].

Several reports in the literature highlight the challenges associated with airway management in the presence of vallecular or epiglottic cysts. Evans reported the inability to insert a laryngeal mask airway (LMA) due to mechanical resistance from a vallecular cyst; aspiration of the cyst restored airway anatomy and enabled continuation of anesthesia [6]. Kariya et al. described a case where ventilation through an intubating LMA (ILMA) failed; fibreoptic bronchoscopy revealed a large epiglottic cyst obscuring the glottic inlet, and intubation was achieved with a fibreoptic bronchoscope only after removing the ILMA and using direct laryngoscopy [7]. These cases reinforce that standard difficult airway algorithms recommending supraglottic airway (SGA) devices as rescue measures may not be reliable in vallecular cysts, where SGA insertion may worsen obstruction, traumatize the cyst, or cause rupture with possible bleeding.

More severe airway compromise has also been documented. Kyle et al. reported a catastrophic “can’t intubate, can’t oxygenate” (CICO) scenario in which both mask ventilation and intubation failed, necessitating emergency surgical tracheostomy [8]. In contrast, our patient had sufficient mask ventilation, which allowed for a controlled bougie-guided intubation with videolaryngoscopy. This emphasizes how crucial it is to have good preparation, receive assistance as soon as possible, be ready for front-of-neck access, and choose the best imaging equipment for children with airway obstruction caused by vallecular cysts.

Our approach was consistent with both the American Society of Anesthesiologists Difficult Airway Algorithm and the All India Difficult Airway Association Paediatric Difficult Airway Guidelines, including early escalation to videolaryngoscopy, maintenance of oxygenation, limitation of repeated laryngoscopy attempts, and early ENT involvement [2,3]. Although SGA devices are generally recommended as rescue options, they were intentionally avoided due to the risk of worsening obstruction, cyst rupture, bleeding, and aspiration associated with vallecular cysts. Table 1 presents the advantages and limitations of the airway management strategy employed in this case.

**Table 1:** Advantages and limitations of the management strategy. ETT = endotracheal tube; SGA = supraglottic airway; ENT = ear, nose, and throat; IV = intravenous

Management decision	Advantage	Limitation
Videolaryngoscopy	Improved glottic view; fewer attempts	Requires expertise and availability
Bougie-guided intubation	Facilitated ETT placement beyond the cyst	Risk of cyst trauma
Avoidance of SGA	Prevented cyst rupture/aspiration	Reduced rescue options
ENT aspiration of the cyst	Definitive airway relief	Requires ENT support
Abandoning planned surgery	Prioritized patient safety	Delayed definitive surgery
IV hydrocortisone	Reduced risk of airway edema	Limited direct evidence

Other examples in the literature elaborate on different approaches. An adult with a massive vallecular cyst who needed a pre-induction tracheostomy because of nearly total airway obstruction was described by Torun et al. [9]. A tongue depressor was successfully used to laterally displace the cyst, allowing for bougie-guided intubation, according to Monem et al. [10]. Tunç et al. experienced unanticipated difficulty with double-lumen tube placement due to a vallecular cyst in an adult, where a pediatric bougie permitted effective intubation [11]. Sawhney et al. described ProSeal LMA failure from an aryepiglottic/vallecular cyst, requiring blind bougie-guided intubation [12].

When combined, these data show that vallecular cysts can make SGA rescue methods and direct laryngoscopy ineffective. Depending on cyst size, airway anatomy, and operator skill, potential alternatives include paraglossal laryngoscopy with a Miller blade, flexible fibreoptic bronchoscopy, videolaryngoscopy, and even retrograde intubation. Crucially, administering preventive steroids, as we did, may help lessen airway edema after challenging or frequent instrumentation.

Therefore, the following principles constitute the basic recommendations for managing unexpected difficult intubation caused by vallecular cysts. First, priority should be given to maintaining adequate oxygenation while simultaneously reducing the number of intubation attempts, as multiple unsuccessful attempts can increase the risk of airway-related complications. Second, early use of advanced airway techniques is advised. Use of videolaryngoscopy or flexible fibreoptic bronchoscopy can improve visualization of the glottic opening and facilitate safer tracheal intubation compared with repeated conventional direct laryngoscopy. Third, blind insertion of SGA devices should be avoided. Vallecular cysts can obstruct proper placement, worsen airway distortion, or rupture during blind advancement, which can lead to complete airway obstruction or aspiration. Fourth, early involvement of ENT support should be considered. Definitive management of the vallecular cyst, such as aspiration or surgical excision, is necessary to secure the airway safely and prevent recurrence of airway compromise. Finally, the airway team must be prepared to secure the airway surgically in such emergencies.

## Conclusions

Our case highlights that although vallecular cysts are uncommon, they should be considered whenever a child’s laryngeal inlet is unexpectedly obscured. Early recognition and selection of an appropriate airway technique are crucial, as conventional strategies such as LMA insertion may fail. Awareness of such airway anomalies is essential for safe pediatric anesthesia.
